# Supplementary material for: Microbiological Hazards in Dry Dog Chews and Feeds
Source: Animals (Basel). 2021 Feb 27;11(3):631. doi: 10.3390/ani11030631 (PMC7997464; doi:10.3390/ani11030631)
Supplement: Supplementary file 1 [file animals-11-00631-s001.pdf]

animals

Supplementary Materials

Microbiological Hazards in Dry Dog Chews and Feeds

Jagoda Kępińska-Pacelik [1], Wioletta Biel [1*]

[1] Department of Monogastric Animal Sciences, Division of Animal Nutrition and Food, West Pomeranian University of Technology in Szczecin, Klemensa Janickiego 29, 71-270 Szczecin, Poland; e-mail: wioletta.biel@zut.edu.pl, orcid.org/0000-0002-3385-6281; e-mail: jagoda.kepinskapacelik@gmail.com, orcid.org/0000-0001-5925-0694

* Correspondence: wioletta.biel@zut.edu.pl

Copyright: © 2021 by the authors. Licensee MDPI, Basel, Switzerland. This article is an open access article distributed under the terms and conditions of the Creative Commons Attribution (CC BY) license (http://creativecommons.org/licenses/by/4.0/).

Table S1. RASFF notifications on pathogenic microorganisms in pet foods products in 2017 to 2020.

**No.**	**Notification type**	**Notifying country**	**Product categories**	**Microorganism**	**Countries concerned by the notification [Origin country]**	**Risk decision**
1.	alert	Germany	dog chews	*Salmonella*	Poland	serious
2.	alert	Germany	dog chews	*Salmonella enterica* ser. Derby	Germany	undecided
3.	information	Sweden	dog chews	*Salmonella*	India	serious
4.	information	Italy	pet food	*Salmonella enterica* ser. Ohio	Spain	not serious
5.	alert	Austria	dog chews	*Salmonella enterica* ser. Rissen, *Salmonella enterica* ser. Typhimurium	Germany	serious
6.	information	Czech Republic	lamb meal	*Salmonella enterica* ser. Bovismorbificans, *Salmonella enterica* ser. Mishmarhaemek	United Kingdom	not serious
7.	alert	United Kingdom	frozen minced beef for dogs	*Salmonella enterica* ser. Paratyphi B (variant Java)	United Kingdom	serious
8.	information	Croatia	frozen pet food	*Salmonella*	United Kingdom	not serious

9.	information	Italy	processed animal proteins	*Salmonella*	Spain	not serious
10.	border rejection	Germany	dog chews	*Salmonella*	Brazil	serious
11.	border rejection	Germany	dog chews (dried tripes)	*Salmonella enterica* ser. Newport, *Salmonella enterica* ser. Typhimurium	India	serious
12.	alert	Germany	dog chews	*Salmonella*	Poland	serious
13.	alert	Belgium	raw petfood	*Salmonella enterica* ser. Typhimurium, *Enterobacteriaceae*	Netherlands	serious
14.	information	Poland	dog chews (dried boar ears)	*Salmonella*	Poland	serious
15.	alert	Norway	frozen raw pet food	*Salmonella enterica* ser. Derby	United Kingdom	serious
16.	information	Belgium	frozen raw pet food	*Salmonella enterica* ser. Onderstepoort, *Enterobacteriaceae*	Netherlands	not serious
17.	information	Poland	pet food	Salmonella	Poland	not serious
18.	alert	Austria	dog chews	*Salmonella enterica* ser. Johannesburg	Germany	serious
19.	alert	Slovenia	frozen raw pet food	*Salmonella enterica* ser. Agona, *Salmonella enterica* ser. Derby	Netherlands	serious
20.	alert	Austria	dog chews	*Salmonella enterica* ser. Infantis, *Enterobacteriaceae*	Germany	serious
21.	information	Croatia	frozen sausages for dogs	*Salmonella*	Croatia	not serious
22.	alert	Austria	dog chews	*Salmonella enterica* ser. Infantis	Poland	serious
23.	alert	Germany	dog chews	*Salmonella*	Netherlands	serious
24.	information	Netherlands	pet food	*Salmonella enterica* ser. Gaminara	Thailand	not serious
25.	alert	Austria	chewing sticks	*Salmonella enterica* ser. Brandenburg, *Salmonella enterica*	Poland	serious

				ser. London, *Salmonella enterica* ser. Rissen, *Salmonella enterica* ser. Typhimurium monophasic		
26.	alert	Austria	dog chews	*Salmonella enterica* ser. Derby	Poland	serious
27.	alert	Austria	dog chews	*Salmonella enterica* ser. Livingstone	Switzerland	serious
28.	alert	Italy	dog chews	*Salmonella enterica* ser. Mapo	Poland	serious
29.	alert	Italy	dog chews	*Salmonella enterica* ser. Derby	Poland	serious
30.	alert	Austria	dog chews	*Salmonella enterica* ser. Mbandaka, *Salmonella enterica* ser. Typhimurium monophasic	Switzerland	serious
31.	alert	Austria	dog chews	*Salmonella enterica* ser. Indiana	Poland	serious
32.	alert	Austria	dog chews	*Salmonella enterica* ser. anatum	Poland	serious
33.	information	Germany	raw pet food	*Salmonella*	Germany	undecided
34.	alert	Norway	frozen raw petfood	*Salmonella enterica* ser. Typhimurium	United Kingdom	serious
35.	alert	Belgium	dog chews	Salmonella enterica ser. Brandenburg, *Salmonella enterica* ser. Idikan, *Enterobacteriaceae*	Netherlands	serious
36.	information	Sweden	pet food	*Salmonella enterica* ser. Derby, *Salmonella enterica* ser. Dublin, *Salmonella enterica* ser. Typhimurium	Germany	not serious
37.	border rejection	Germany	dog chews	*Salmonella, Enterobacteriaceae*	Turkey	serious
38.	information	Belgium	raw petfood	*Salmonella, Enterobacteriaceae*	Netherlands	not serious

39.	alert	Austria	dog chews	*Salmonella enterica* ser. Agona	Turkey	serious
40.	alert	Austria	dog chews	*Salmonella enterica* ser. Derby, *Salmonella enterica* ser. Typhimurium monophasic	Belgium	serious
41.	alert	Austria	dog chews	*Salmonella* enterica ser. Hessarek	Germany	serious
42.	alert	Austria	dried pig ears	*Salmonella enterica* ser. Derby, *Salmonella enterica* ser. Livingstone	Germany	serious
43.	information	Germany	dog chews	*Salmonella*	France	serious
44.	alert	Austria	dried chicken feet	*Salmonella enterica* ser. Münster	Poland	serious
45.	alert	Austria	chicken feet	*Salmonella enterica* ser. Gold Coast, *Enterobacteriaceae*	Poland	serious
46.	alert	Austria	dog chews	*Salmonella enterica* ser. Indiana, *Enterobacteriaceae*	Poland	serious
47.	border rejection	Germany	dog chew (lamb ears)	*Salmonella*	Turkey	serious
48.	alert	Austria	dog chews (bovine scalp and throat)	*Salmonella enterica* ser. Infantis	Slovakia	serious
49.	information	Belgium	frozen raw dog food	*Salmonella enterica* ser. Indiana, *Enterobacteriaceae*	Netherlands	not serious
50.	alert	Austria	dog chews	*Salmonella enterica* ser. Derby, *Salmonella enterica* ser. Typhimurium monophasic	Poland	serious
51.	information	Poland	dog chews	*Salmonella*	Turkey	serious
52.	border rejection	Germany	dog food	*Salmonella enterica* ser. anatum	Mexico	serious
53.	information	Belgium	frozen dog food	*Salmonella, Enterobacteriaceae*	Netherlands	not serious

54.	alert	Germany	dog chews	*Salmonella*	India	serious
55.	alert	Germany	dog chews	*Salmonella, Enterobacteriaceae*	Turkey	serious
56.	information	Belgium	raw dog food	*Salmonella enterica* ser. Derby, *Salmonella enterica* ser. Mbandaka, *Enterobacteriaceae*	Netherlands	not serious
57.	alert	Germany	dog chews	*Salmonella*	Poland	serious
58.	alert	Sweden	dog chews	*Salmonella enterica* ser. Senftenberg	Poland	serious
59.	alert	Belgium	dog chews from camel and horse	*Salmonella* group E1	Poland	serious
60.	alert	Italy	dried cow's liver	*Salmonella*	Italy	serious
61.	alert	Austria	dog chews	*Salmonella enterica* ser. Derby	Poland	serious
62.	border rejection	Netherlands	dog chews	*Salmonella*	Thailand	serious
63.	border rejection	Netherlands	dog chews	*Salmonella*	Thailand	serious
64.	alert	Austria	dog chews	*Salmonella enterica* ser. Infantis	Netherlands	serious
65.	alert	Lithuania	dog chews	*Salmonella enterica* ser. Derby, *Salmonella enterica* ser. Enteritidis, *Salmonella enterica* ser. London	Belarus	serious
66.	alert	Austria	dog chews	*Salmonella enterica* ser. Derby, *Salmonella enterica* ser. Livingstone, *Salmonella enterica* ser. London	Germany	serious

Source: own study on the basis of [82]
